# Author Correction: Structural determinants and functional consequences of protein affinity for membrane rafts

**DOI:** 10.1038/s41467-018-04164-1

**Published:** 2018-05-01

**Authors:** Joseph H. Lorent, Blanca Diaz-Rohrer, Xubo Lin, Kevin Spring, Alemayehu A. Gorfe, Kandice R. Levental, Ilya Levental

**Affiliations:** McGovern Medical School, University of Texas Health Science Center, Houston MSB 4.202A, 6431 Fannin St, 77096 Houston, TX USA

**Correction to**: *Nature Communications* 10.1038/s41467-017-01328-3; published online 31 October 2017

In the originally published version of this Article, financial support was not fully acknowledged. The PDF and HTML versions of the Article have now been corrected to include support from National Institute of General Medical Sciences, National Institutes of Health grant R01GM124072.

