# Supplementary material for: Structural determinants and functional consequences of protein affinity for membrane rafts
Source: Nat Commun. 2017 Oct 31;8:1219. doi: 10.1038/s41467-017-01328-3 (PMC5663905; doi:10.1038/s41467-017-01328-3)
Supplement: Supplementary file 1 — Supplementary Information [file 41467_2017_1328_MOESM1_ESM.pdf]

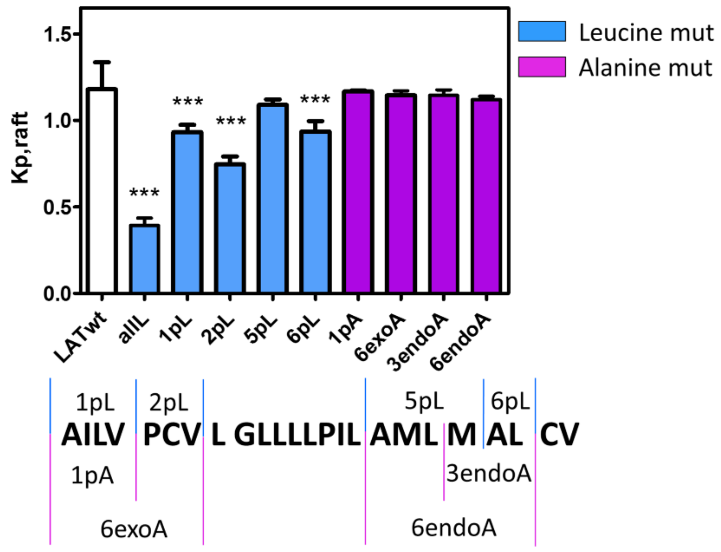

Supplementary Figure 1. Leu mutations are more disruptive of raft partitioning than Ala mutations. Raft partitioning for various Ala (purple) or Leu (blue) mutations of the LAT TMD. Along the whole length of the TMD, Ala mutations of up to 6 residues had no effect on partitioning, whereas Leu mutations of similar residues often inhibited raft association.Average +/- SD for 3-5 independent trials, each with >10 vesicles/condition. Significances are one-way ANOVA relative to wild-type LAT; $^{***}p<0.001$; $^{**}p<0.01$.

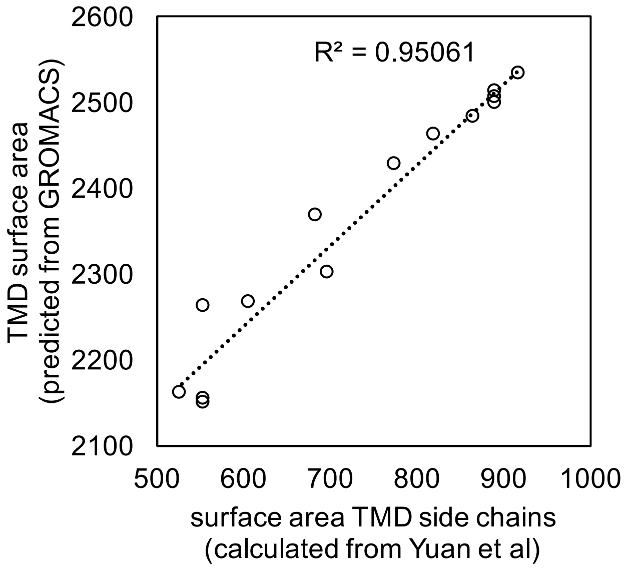

Supplementary Figure 2. Validation of predicted surface area by computational modeling. Correlation ($p<0.0001$) between TMD side chain surface area calculated from values in Yuan et al [1] and TMD surface area calculated by computational modeling in GROMACS for 14 representative TMDs. The values from computational modeling are much larger because this method measures the surface area of the TMD as a whole, rather than only the side chains. Nevertheless, the excellent correlation suggests that both methods are effective estimates of surface area trends.

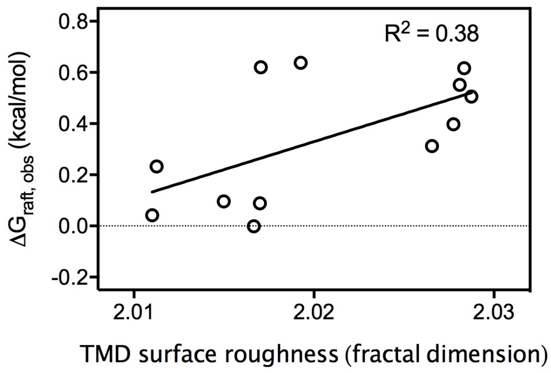

Supplementary Figure 3. Weak correlation between raft affinity and TMD surface roughness. TMD roughness is calculated via the fractal dimension for probes between 5-6 Å. This length scale was chosen because it represent the persistence length of a lipid acyl chain in a fluid bilayer, previously approximated at ~5 Å[2]. At this scale, all TMDs tested appear nearly smooth (fractal dimension = 2 for an ideal smooth surface).

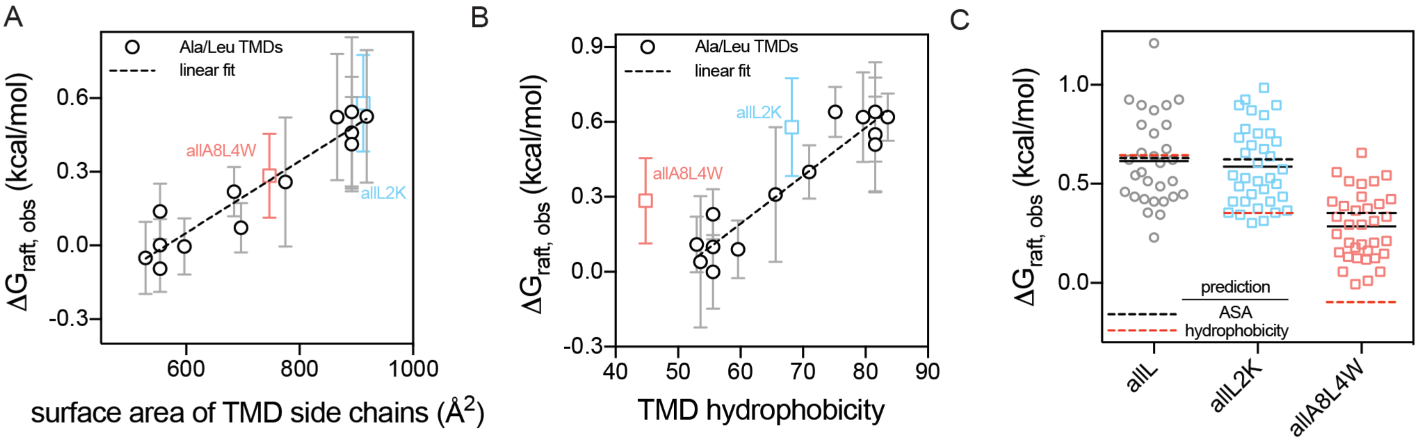

Supplementary Figure 4. TMD surface area rather than hydrophobicity is a determinant of raft affinity. (A) Strong correlation between TMD surface area and raft affinity established for TMDs consisting only of Ala and Leu (black circles; also shown as Fig 2C) also holds for TMDs containing charged (allL2K; blue square) or hydrophilic residues (allA8L4W; red square). (B) The raft affinity of these hydrophilic residue-containing TMDs is not well described by their aggregate hydrophobicity (calculated via the scale of Kyte and Doolittle [3]). (C) Scatter plot showing raft affinity measurements for individual vesicles and the predictions from the linear regressions shown in panels A-B.

**allL2K**	M	E	E	L	L	L	L	L	L	L	L	K	L	L	L	L	L	K	L	L	L	L	L	L	L	C	V	H	C
**allA8L4W**	M	E	E	L	A	A	L	A	A	L	A	W	A	L	A	W	L	A	W	L	A	W	L	A	L	C	V	H	C

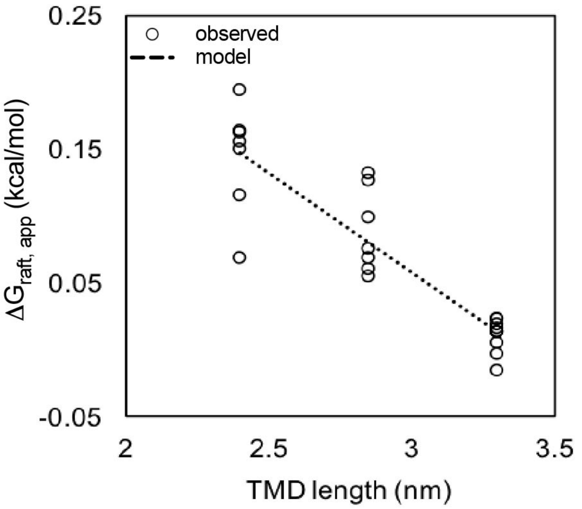

Supplementary Figure 5. Fitting partitioning as a function of TMD length to simplified mattress model. Published data on partitioning of TMDs of varying lengths[4] was fitted with a simplified version of the Mouritsen hydrophobic mismatch "mattress" model [5]: $\Delta G_{raft,app} = -2B_{LP} (L_{TMD} - 0.5 (L_{Lo} + L_{Ld}))$. The thicknesses of the coexisting L_o (3.9 nm) and L_d (3.6 nm) phases were estimated from analysis of published all-atom simulations of phase separated membranes [6] (personal communication Dr Edward Lyman, University of Delaware). L_{TMD} was estimated from the number of hydrophobic amino acids (predicted by TMHMM[7]) multiplied by 1.5 Å/residue for an α-helix. B_{LP} was the only fit parameter ($7.5x10^{-2}$ kcal/mol.nm), which agrees well with previous estimates [8].

For the effect of palmitoylation, we used previously measurements of raft partitioning of the palmitoylation mutants of the LAT TMD (i.e. C26A and C29A) [9]. The $K_{p,raft}$ values for the two palmitoylation mutants were converted to $\Delta G_{raft,app}$ ($\Delta G_{raft,app} = -RT \ln K_{p,raft}$; values in Supp Table III), then averaged to yield the expected effect of palmitoylation of raft affinity (-0.48 kcal/mol/palmitoylation).

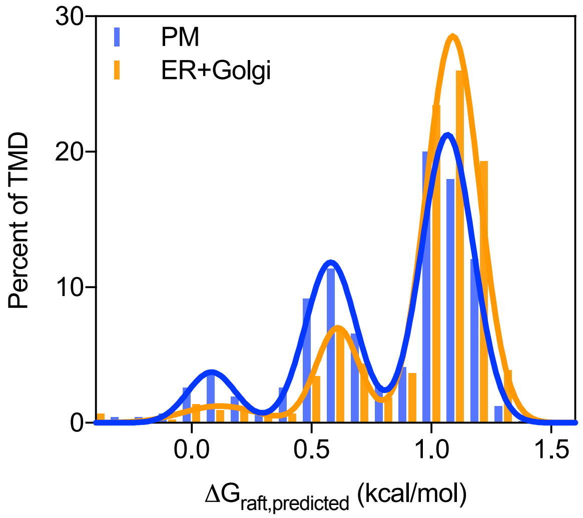

Supplementary Figure 6. Histogram of predicted raft partitioning values from Fig 4D. Predicted PM TMDs are shown in blue, ER and Golgi TMDs are in orange.

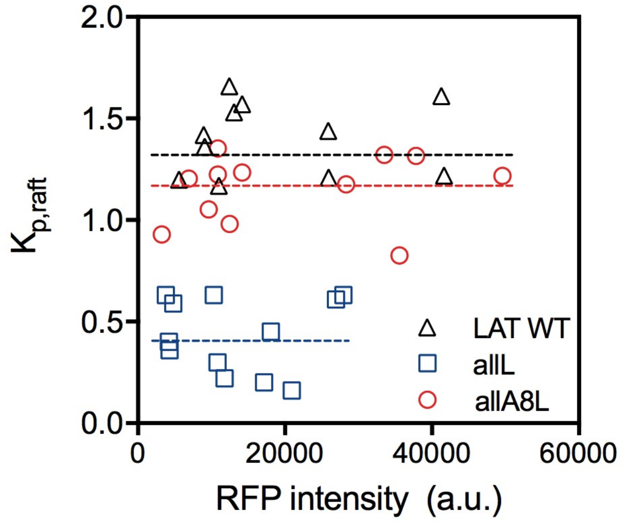

Supplementary Figure 7. No notable correlation observed between expression level and raft partitioning. No correlation was observed between raft partitioning and PM expression level (RFP intensity in GPMVs used as a proxy for PM expression) for either raft- or non-raft-preferring TMDs.

Supplementary Table 1. Sequences and raft partitioning values of LAT TMD variants used for Figure 1.

Name	1	2	3	4	5	6	7	8	9	10	11	12	13	14	15	16	17	18	19	20	21	22	23	24	25	26		Kp,raft	StDev Kp	△Graft,app	StDev △G
**wild type**	M	E	E	A	I	L	V	P	C	V	L	G	L	L	L	L	P	I	L	A	M	L	M	A	L	C	…	1.00	0.16	0.00	0.09
**allL**	M	E	E	**L**	**L**	L	**L**	**L**	**L**	**L**	L	**L**	L	L	L	L	**L**	**L**	L	**L**	**L**	L	**L**	**L**	L	C	…	0.33	0.16	0.62	0.27
**1pL**	M	E	E	**L**	**L**	L	**L**	P	C	V	L	G	L	L	L	L	P	I	L	A	M	L	M	A	L	C	…	0.79	0.16	0.13	0.11
**2pL**	M	E	E	A	I	L	V	**L**	**L**	**L**	L	G	L	L	L	L	P	I	L	A	M	L	M	A	L	C	…	0.63	0.17	0.26	0.15
**3pL (G12L)**	M	E	E	A	I	L	V	P	C	V	L	**L**	L	L	L	L	P	I	L	A	M	L	M	A	L	C	…	0.84	0.25	0.10	0.17
**4pL**	M	E	E	A	I	L	V	P	C	V	L	G	L	L	L	L	**L**	**L**	L	A	M	L	M	A	L	C	…	0.91	0.16	0.06	0.10
**5pL**	M	E	E	A	I	L	V	P	C	V	L	G	L	L	L	L	P	I	L	**L**	**L**	L	**L**	A	L	C	…	0.93	0.12	0.04	0.07
**6pL**	M	E	E	A	I	L	V	P	C	V	L	G	L	L	L	L	P	I	L	A	M	L	M	**L**	L	C	…	0.79	0.23	0.13	0.16
**scr2**	M	E	E	A	I	L	V	**L**	C	**P**	L	G	L	**A**	**I**	L	**L**	**V**	**M**	**L**	M	**P**	**L**	A	L	C	…	0.98	0.19	0.01	0.11

Red text denotes the location of the mutations from the wild-type LAT TMD.

Supplementary Table 2. Sequences and raft affinities of LAT TMD variants used for Figure 2.

Name	1	2	3	4	5	6	7	8	9	10	11	12	13	14	15	16	17	18	19	20	21	22	23	24	25	26		△Graft,app	StDev △G	ASA
6coreA	M	E	E	A	I	L	V	P	C	V	L	G	A	A	A	A	A	A	L	A	M	L	M	A	L	C	…	-0.02	0.17	566.66
6exoA	M	E	E	A	A	A	A	A	A	A	L	G	L	L	L	L	P	I	L	A	M	L	M	A	L	C	…	0.02	0.06	605.28
9exoA	M	E	E	A	A	A	A	A	A	A	A	A	A	L	L	L	P	I	L	A	M	L	M	A	L	C	…	0.00	0.08	559.90
allA+3L4L	M	E	E	L	A	A	A	L	A	L	A	L	A	A	A	A	L	A	A	A	A	L	A	A	L	C	…	0.04	0.15	528.51
allA+8L	M	E	E	L	A	A	L	A	A	L	A	A	L	A	A	L	A	A	L	A	A	L	A	A	L	C	…	0.00	0.10	554.48
allL+3G+A	M	E	E	L	L	A	L	G	L	L	A	G	A	A	A	A	G	L	A	L	L	A	L	L	A	C	…	0.11	0.18	586.74
allL+A	M	E	E	L	L	A	L	L	L	L	A	L	A	A	A	A	L	L	A	L	L	A	L	L	A	C	…	0.31	0.10	684.33
allL+3A+A	M	E	E	L	L	A	L	A	L	L	A	A	A	A	A	A	A	L	A	L	L	A	L	L	A	C	…	0.09	0.11	606.42
allL+3G	M	E	E	L	L	L	L	G	L	L	L	G	L	L	L	L	G	L	L	L	L	L	L	L	L	C	…	0.40	0.21	820.47
allL+A12	M	E	E	L	L	L	L	L	L	L	L	A	L	L	L	L	L	L	L	L	L	L	L	L	L	C	…	0.55	0.23	892.09
allL+A17	M	E	E	L	L	L	L	L	L	L	L	L	L	L	L	L	A	L	L	L	L	L	L	L	L	C	…	0.64	0.30	892.09
allL+A8	M	E	E	L	L	L	L	A	L	L	L	L	L	L	L	L	L	L	L	L	L	L	L	L	L	C	…	0.51	0.19	892.09
allL+A8/A12	M	E	E	L	L	L	L	A	L	L	L	A	L	L	L	L	L	L	L	L	L	L	L	L	L	C	…	0.62	0.26	866.12
allL+C9/G12	M	E	E	L	L	L	L	L	C	L	L	G	L	L	L	L	L	L	L	L	L	L	L	L	L	C	…	0.62	0.27	855.60
allL+G8/G12	M	E	E	L	L	L	L	G	L	L	L	G	L	L	L	L	L	L	L	L	L	L	L	L	L	C	…	0.64	0.22	853.00
LinAout	M	E	E	A	A	A	A	A	A	A	L	L	L	L	L	L	L	L	A	A	A	A	A	A	A	C	…	0.23	0.11	554.48
LoutAin	M	E	E	L	L	L	L	A	A	A	A	A	A	A	A	A	A	A	A	A	A	L	L	L	L	C	…	0.10	0.11	554.48
manyL+P8/P17	M	E	E	A	I	L	V	P	L	L	L	L	L	L	L	L	P	L	L	L	L	L	L	L	L	C	…	0.72	0.38	834.32
manyL+P17	M	E	E	A	I	L	V	L	L	L	L	L	L	L	L	L	P	L	L	L	L	L	L	L	L	C	…	0.61	0.31	857.36
switchLA+G12/P17	M	E	E	L	L	A	L	L	L	L	A	G	A	A	A	A	P	L	A	L	L	A	L	L	A	C	…	0.07	0.12	628.76
P7P18	M	E	E	A	I	L	P	V	C	V	L	G	L	L	L	L	I	P	L	A	M	L	M	A	L	C	…	0.01	0.12	696.44
scr2	M	E	E	A	I	L	V	L	C	P	L	G	L	A	I	L	L	V	M	L	M	P	L	A	L	C	…	0.05	0.19	696.44
P8P17G	M	E	E	A	I	L	V	G	C	V	L	G	L	L	L	L	G	I	L	A	M	L	M	A	L	C	…	-0.03	0.26	677.46
C9/G12L	M	E	E	A	I	L	V	P	L	V	L	L	L	L	L	L	P	I	L	A	M	L	M	A	L	C	…	0.22	0.13	750.21
3qS	M	E	E	A	I	L	V	P	C	V	L	V	C	L	G	L	P	I	L	A	M	L	M	A	L	C	…	0.14	0.21	666.51
4qS	M	E	E	A	I	L	V	P	C	V	L	G	L	L	L	P	M	L	A	A	M	L	M	A	L	C	…	-0.01	0.10	663.23
allL	M	E	E	L	L	L	L	L	L	L	L	L	L	L	L	L	L	L	L	L	L	L	L	L	L	C	…	0.62	0.27	918.06
2pL	M	E	E	A	I	L	V	L	L	L	L	G	L	L	L	L	P	I	L	A	M	L	M	A	L	C	…	0.26	0.15	749.41
3pL (G12L)	M	E	E	A	I	L	V	P	C	V	L	L	L	L	L	L	P	I	L	A	M	L	M	A	L	C	…	0.10	0.17	728.97
4pL	M	E	E	A	I	L	V	P	C	V	L	G	L	L	L	L	L	L	L	A	M	L	M	A	L	C	…	0.06	0.10	722.48
P8/G12/P17L	M	E	E	A	I	L	V	L	C	V	L	L	L	L	L	L	L	I	L	A	M	L	M	A	L	C	…	0.35	0.26	775.05
P8/G12/P17A	M	E	E	A	I	L	V	A	C	V	L	A	L	L	L	L	A	I	L	A	M	L	M	A	L	C	…	0.07	0.12	697.14
allL+P8/P17	M	E	E	L	L	L	L	P	L	L	L	L	L	L	L	L	P	L	L	L	L	L	L	L	L	C	…	0.59	0.16	871.98
allL+P8/G12	M	E	E	L	L	L	L	P	L	L	L	G	L	L	L	L	L	L	L	L	L	L	L	L	L	C	…	0.47	0.14	862.49
allL+G12/P17	M	E	E	L	L	L	L	L	L	L	L	G	L	L	L	L	P	L	L	L	L	L	L	L	L	C	…	0.47	0.22	862.49
P8/P17L	M	E	E	A	I	L	V	L	C	V	L	G	L	L	L	L	L	I	L	A	M	L	M	A	L	C	…	0.48	0.20	742.52
P8/P17A	M	E	E	A	I	L	V	A	C	V	L	G	L	L	L	L	A	I	L	A	M	L	M	A	L	C	…	0.15	0.26	690.58
P8/G12L	M	E	E	A	I	L	V	L	C	V	L	L	L	L	L	L	P	I	L	A	M	L	M	A	L	C	…	0.29	0.14	752.01
G12/P17L	M	E	E	A	I	L	V	P	C	V	L	L	L	L	L	L	L	I	L	A	M	L	M	A	L	C	…	0.49	0.20	752.01
P8/P17F	M	E	E	A	I	L	V	F	C	V	L	G	L	L	L	L	F	I	L	A	M	L	M	A	L	C	…	0.25	0.11	749.44
P8/P17I	M	E	E	A	I	L	V	I	C	V	L	G	L	L	L	L	I	I	L	A	M	L	M	A	L	C	…	0.50	0.56	736.52
P8L	M	E	E	A	I	L	V	L	C	V	L	G	L	L	L	L	P	I	L	A	M	L	M	A	L	C	…	0.15	0.15	719.48
P8A	M	E	E	A	I	L	V	A	C	V	L	G	L	L	L	L	P	I	L	A	M	L	M	A	L	C	…	0.10	0.17	693.51
G12A	M	E	E	A	I	L	V	P	C	V	L	A	L	L	L	L	P	I	L	A	M	L	M	A	L	C	…	0.06	0.08	703.00
P17L	M	E	E	A	I	L	V	P	C	V	L	G	L	L	L	L	L	I	L	A	M	L	M	A	L	C	…	0.09	0.16	719.48
P17A	M	E	E	A	I	L	V	P	C	V	L	G	L	L	L	L	A	I	L	A	M	L	M	A	L	C	…	0.14	0.15	693.51
G12A/P17L	M	E	E	A	I	L	V	P	C	V	L	A	L	L	L	L	L	I	L	A	M	L	M	A	L	C	…	-0.01	0.14	726.04
P8L/G12A	M	E	E	A	I	L	V	L	C	V	L	A	L	L	L	L	P	I	L	A	M	L	M	A	L	C	…	0.02	0.11	726.04

Red text denotes the location of the mutations from the wild-type LAT TMD.

Supplementary Table 3. Sequences and observed and predicted raft affinities of TMD variants used for Figure 3B and S4.

Name	1	2	3	4	5	6	7	8	9	10	11	12	13	14	15	16	17	18	19	20	21	22	23	24	25	26	27	28	29	△Graft,app	△Graft,pred
1pL	M	E	E	L	L	L	L	P	C	V	L	G	L	L	L	L	P	I	L	A	M	L	M	A	L	C	V	H	C	0.26	0.29
1qS	M	E	E	A	I	L	V	I	L	M	L	G	L	L	L	L	P	I	L	A	M	L	M	A	L	C	V	H	C	0.02	0.27
P8P17A	M	E	E	A	I	L	V	A	C	V	L	G	L	L	L	L	A	I	L	A	M	L	M	A	L	C	V	H	C	0.15	0.20
P8P17F	M	E	E	A	I	L	V	F	C	V	L	G	L	L	L	L	F	I	L	A	M	L	M	A	L	C	V	H	C	0.25	0.29
P8G12P17L	M	E	E	A	I	L	V	L	C	V	L	L	L	L	L	L	L	I	L	A	M	L	M	A	L	C	V	H	C	0.35	0.33
P8P17I	M	E	E	A	I	L	V	I	C	V	L	G	L	L	L	L	I	I	L	A	M	L	M	A	L	C	V	H	C	0.50	0.27
P8P17L	M	E	E	A	I	L	V	L	C	V	L	G	L	L	L	L	L	I	L	A	M	L	M	A	L	C	V	H	C	0.48	0.28
2qS	M	E	E	A	I	L	V	P	C	V	L	V	C	L	G	L	P	I	L	A	M	L	M	A	L	C	V	H	C	0.14	0.16
3pL	M	E	E	A	I	L	V	P	C	V	L	L	L	L	L	L	P	I	L	A	M	L	M	A	L	C	V	H	C	0.06	0.25
3qS	M	E	E	A	I	L	V	P	C	V	L	G	L	L	L	P	M	L	A	A	M	L	M	A	L	C	V	H	C	-0.01	0.15
6coreA	M	E	E	A	I	L	V	P	C	V	L	G	A	A	A	A	A	A	L	A	M	L	M	A	L	C	V	H	C	-0.02	0.00
6exoA	M	E	E	A	A	A	A	A	A	A	L	G	L	L	L	L	P	I	L	A	M	L	M	A	L	C	V	H	C	0.02	0.06
9exoA	M	E	E	A	A	A	A	A	A	A	A	A	A	L	L	L	P	I	L	A	M	L	M	A	L	C	V	H	C	0.00	-0.01
allA+3L4L	M	E	E	L	A	A	A	L	A	L	A	L	A	A	A	A	L	A	A	A	A	L	A	A	L	C	V	H	C	0.04	-0.06
allA+8L	M	E	E	L	A	A	L	A	A	L	A	A	L	A	A	L	A	A	L	A	A	L	A	A	L	C	V	H	C	0.00	-0.02
allA8L_C1A	M	E	E	L	A	A	L	A	A	L	A	A	L	A	A	L	A	A	L	A	A	L	A	A	L	A	V	H	C	0.41	0.46
allA8L_C2A	M	E	E	L	A	A	L	A	A	L	A	A	L	A	A	L	A	A	L	A	A	L	A	A	L	C	V	H	A	0.14	0.46
allL	M	E	E	L	L	L	L	L	L	L	L	L	L	L	L	L	L	L	L	L	L	L	L	L	L	C	V	H	C	0.62	0.56
allL_3GnLA	M	E	E	L	L	A	L	G	L	L	A	G	A	A	A	A	G	L	A	L	L	A	L	L	A	C	V	H	C	0.11	0.03
allL_nLA	M	E	E	L	L	A	L	L	L	L	A	L	A	A	A	A	L	L	A	L	L	A	L	L	A	C	V	H	C	0.31	0.19
allLP8P17	M	E	E	L	L	L	L	P	L	L	L	L	L	L	L	L	P	L	L	L	L	L	L	L	L	C	V	H	C	0.59	0.48
allL3A_nLA	M	E	E	L	L	A	L	A	L	L	A	A	A	A	A	A	A	L	A	L	L	A	L	L	A	C	V	H	C	0.09	0.06
allL3G	M	E	E	L	L	L	L	G	L	L	L	G	L	L	L	L	G	L	L	L	L	L	L	L	L	C	V	H	C	0.40	0.40
allLA12	M	E	E	L	L	L	L	L	L	L	L	A	L	L	L	L	L	L	L	L	L	L	L	L	L	C	V	H	C	0.55	0.52
allLA17	M	E	E	L	L	L	L	L	L	L	L	L	L	L	L	L	A	L	L	L	L	L	L	L	L	C	V	H	C	0.64	0.52
allLA8	M	E	E	L	L	L	L	A	L	L	L	L	L	L	L	L	L	L	L	L	L	L	L	L	L	C	V	H	C	0.51	0.52
allLA8A12	M	E	E	L	L	L	L	A	L	L	L	A	L	L	L	L	L	L	L	L	L	L	L	L	L	C	V	H	C	0.62	0.47
allLC9G12	M	E	E	L	L	L	L	L	C	V	L	G	L	L	L	L	L	L	L	L	L	L	L	L	L	C	V	H	C	0.62	0.46
allLG8G12	M	E	E	L	L	L	L	G	L	L	L	G	L	L	L	L	L	L	L	L	L	L	L	L	L	C	V	H	C	0.64	0.45
allLG12P17	M	E	E	L	L	L	L	L	L	L	L	G	L	L	L	L	P	L	L	L	L	L	L	L	L	C	V	H	C	0.47	0.47
allLP8G12	M	E	E	L	L	L	L	P	L	L	L	G	L	L	L	L	L	L	L	L	L	L	L	L	L	C	V	H	C	0.47	0.47
G12A	M	E	E	A	I	L	V	P	C	V	L	A	L	L	L	L	P	I	L	A	M	L	M	A	L	C	V	H	C	0.06	0.22
G12L	M	E	E	A	I	L	V	P	C	V	L	L	L	L	L	L	P	I	L	A	M	L	M	A	L	C	V	H	C	0.10	0.26
G12P17L	M	E	E	A	I	L	V	P	C	V	L	L	L	L	L	L	L	I	L	A	M	L	M	A	L	C	V	H	C	0.49	0.29
LATd3core	M	E	E	A	I	L	V	P	C	V	L	G	L	-	-	-	P	I	L	A	M	L	M	A	L	C	V	H	C	0.07	0.07
LATd3exo	M	E	E	-	-	-	V	P	C	V	L	G	L	L	L	L	P	I	L	A	M	L	M	A	L	C	V	H	C	0.17	0.12
LATd6core	M	E	E	A	I	L	V	P	C	V	L	G	L	-	-	-	-	-	-	A	M	L	M	A	L	C	V	H	C	0.23	-0.02
LATd6exo	M	E	E	-	-	-	-	-	-	V	L	G	L	L	L	L	P	I	L	A	M	L	M	A	L	C	V	H	C	0.09	0.07
LinAout	M	E	E	A	A	A	A	A	A	A	L	L	L	L	L	L	L	L	A	A	A	A	A	A	A	C	V	H	C	0.23	0.00
LoutAin	M	E	E	L	L	L	L	A	A	A	A	A	A	A	A	A	A	A	A	A	A	L	L	L	L	C	V	H	C	0.10	-0.02
mLP8P17	M	E	E	A	I	L	V	P	L	L	L	L	L	L	L	L	P	L	L	L	L	L	L	L	L	C	V	H	C	0.72	0.42
mLP17	M	E	E	A	I	L	V	L	L	L	L	L	L	L	L	L	P	L	L	L	L	L	L	L	L	C	V	H	C	0.61	0.46
nLA_P8G12	M	E	E	L	L	A	L	P	L	L	A	G	A	A	A	A	L	L	A	L	L	A	L	L	A	C	V	H	C	0.12	0.10
nLA-G12P17	M	E	E	L	L	A	L	L	L	L	A	G	A	A	A	A	P	L	A	L	L	A	L	L	A	C	V	H	C	0.07	0.10
P17A	M	E	E	A	I	L	V	P	C	V	L	G	L	L	L	L	A	I	L	A	M	L	M	A	L	C	V	H	C	0.14	0.20
P17L	M	E	E	A	I	L	V	P	C	V	L	G	L	L	L	L	L	I	L	A	M	L	M	A	L	C	V	H	C	0.09	0.24
P7P18	M	E	E	A	I	L	P	V	C	V	L	G	L	L	L	L	I	P	L	A	M	L	M	A	L	C	V	H	C	0.01	0.21
P8A	M	E	E	A	I	L	V	A	C	V	L	G	L	L	L	L	P	I	L	A	M	L	M	A	L	C	V	H	C	0.10	0.20
P8G12L	M	E	E	A	I	L	V	L	C	V	L	L	L	L	L	L	P	I	L	A	M	L	M	A	L	C	V	H	C	0.29	0.29
P8L	M	E	E	A	I	L	V	L	C	V	L	G	L	L	L	L	P	I	L	A	M	L	M	A	L	C	V	H	C	0.15	0.24
C26A	M	E	E	A	I	L	V	P	C	V	L	G	L	L	L	L	P	I	L	A	M	L	M	A	L	A	V	H	C	0.76	0.68
C29A	M	E	E	A	I	L	V	P	C	V	L	G	L	L	L	L	P	I	L	A	M	L	M	A	L	C	V	H	A	0.21	0.68
wt_3G	M	E	E	A	I	L	V	G	C	V	L	G	L	L	L	L	G	I	L	A	M	L	M	A	L	C	V	H	C	-0.03	0.18
C9G12L	M	E	E	A	I	L	V	P	L	V	L	L	L	L	L	L	P	I	L	A	M	L	M	A	L	C	V	H	C	0.22	0.29
P8G12P17A	M	E	E	A	I	L	V	A	C	V	L	A	L	L	L	L	A	I	L	A	M	L	M	A	L	C	V	H	C	0.07	0.21
P17LG12A	M	E	E	A	I	L	V	P	C	V	L	A	L	L	L	L	L	I	L	A	M	L	M	A	L	C	V	H	C	-0.01	0.25
P8LG12A	M	E	E	A	I	L	V	L	C	V	L	A	L	L	L	L	P	I	L	A	M	L	M	A	L	C	V	H	C	0.02	0.25

Red text denotes the mutations from the wild-type LAT TMD.

Supplementary Table 4. Sequences and observed raft partitioning values of TMD variants used for Figure 3D-G.

Name	1	2	3	4	5	6	7	8	9	10	11	12	13	14	15	16	17	18	19	20	21	22	23	24	25	26	27	28	29	30	31	32	Kp,raft
**LAT WT**	M	E	E	A	I	L	V	P	C	V	L	G	L	L	L	L	P	I	L	A	M	L	M	A	L	C	V	H	C				1.00
**LAT C26A**	M	E	E	A	I	L	V	P	C	V	L	G	L	L	L	L	P	I	L	A	M	L	M	A	L	**A**	V	H	C				0.76
**LAT C29A**	M	E	E	A	I	L	V	P	C	V	L	G	L	L	L	L	P	I	L	A	M	L	M	A	L	C	V	H	**A**				0.21
**LAT d6exo**	M	E	E	-	-	-	-	-	-	V	L	G	L	L	L	L	P	I	L	A	M	L	M	A	L	C	V	H	C				0.85
**LAT d6core**	M	E	E	A	I	L	V	P	C	V	L	G	L	-	-	-	-	-	-	A	M	L	M	A	L	C	V	H	C				0.66
**LAT high ASA**	M	E	E	A	I	L	V	**L**	**L**	**L**	L	**L**	L	L	L	L	P	**L**	L	**L**	**L**	L	**L**	**L**	L	C	V	H	C				0.32
**PAG WT**	*	M	Q	I	T	L	W	G	S	L	A	A	V	A	I	F	F	V	I	T	F	L	I	F	L	C	S	S	C				0.92
**PAG C37A**	*	M	Q	I	T	L	W	G	S	L	A	A	V	A	I	F	F	V	I	T	F	L	I	F	L	**A**	S	S	C				0.48
**PAG C40A**	*	M	Q	I	T	L	W	G	S	L	A	A	V	A	I	F	F	V	I	T	F	L	I	F	L	C	S	S	**A**				0.66
**PAG d6exo**	*	M	Q	I	T	L	W	-	-	-	-	-	-	A	I	F	F	V	I	T	F	L	I	F	L	C	S	S	C				0.54
**PAG d6core**	*	M	Q	I	T	L	W	G	S	L	A	A	V	-	-	-	-	-	-	T	F	L	I	F	L	C	S	S	C				0.50
**PAG high ASA**	*	M	Q	I	T	L	W	**F**	S	L	**L**	**F**	**L**	**L**	**L**	F	F	**L**	**F**	**L**	F	L	**L**	F	L	C	S	S	C				0.45
**LIME WT**	M	G	L	P	V	S	W	A	P	P	A	L	W	V	L	G	C	C	A	L	L	L	S	L	W	A	L	C	T	A	C		1.06
**LIME d6exo**	M	G	L	P	V	S	W	A	P	-	-	-	-	-	-	G	C	C	A	L	L	L	S	L	W	A	L	C	T	A	C		1.00
**LIME d6core**	M	G	L	P	V	S	W	A	P	P	A	L	W	V	L	-	-	-	-	-	-	L	S	L	W	A	L	C	T	A	C		0.75
**LIME high ASA**	M	G	L	P	V	S	W	A	P	P	**L**	L	**F**	**L**	L	**L**	**F**	C	**L**	L	L	L	S	L	W	**L**	L	C	T	A	C		0.39
**LDLR TMD*** **	*M*	*E*	*E*	A	L	S	I	V	L	P	I	V	L	L	V	F	L	C	L	G	V	F	L	L	W	*C*	*V*	*H*	*C*				0.39
**LDLR low ASA**	*M*	*E*	*E*	A	**A**	S	I	V	**A**	P	**A**	V	**A**	**A**	V	**A**	**A**	C	L	G	V	**A**	**A**	L	W	*C*	*V*	*H*	*C*				0.86
**CD4 TMD*** **	*M*	*E*	*E*	V	Q	P	M	A	L	I	V	L	G	G	V	A	G	L	L	L	F	I	G	L	G	I	F	F	*C*	V	H	C	1.00
**LAX TMD*** **	*M*	*E*	*E*	I	F	S	G	F	A	G	L	L	A	I	L	L	V	V	A	V	F	C	I	L	W	*C*	*V*	*H*	*C*				0.64

Red text denotes the mutations from the relevant wild-type TMD.
*MGPAGSLLGSGQ…
*** LDLR, CD4, and LAX constructs only contain the TMD of these proteins, with the flanking sequences of hLAT (italic font). This was done to ensure proper membrane integration of the TMDs in the absence of their native extracellular domains.

Supplementary methods

Determination of ASA. ASA was calculated in GROMACS by g_sas after a steep descent energy minimization of 50000 steps.

TMD roughness. TMD roughness was computationally calculated via the fractal dimension [10]. Excluded surface area was calculated by the MSMS algorithm [11] using spherical probes between 5-6 Å at 0.1 Å steps. Aggregate TMD hydrophobicity was calculated using the Kyte and Doolittle scale [3].

Supplementary References

1 Yuan, Z. *et al.* Predicting the solvent accessibility of transmembrane residues from protein sequence. *J Proteome Res* **5**, 1063-1070, (2006).
2 Rawicz, W. *et al.* Effect of chain length and unsaturation on elasticity of lipid bilayers. *Biophys J* **79**, 328-339, (2000).
3 Kyte, J. & Doolittle, R. F. A simple method for displaying the hydropathic character of a protein. *Journal of molecular biology* **157**, 105-132, (1982).
4 Diaz-Rohrer, B. B., Levental, K. R., Simons, K. & Levental, I. Membrane raft association is a determinant of plasma membrane localization. *Proc Natl Acad Sci U S A* **111**, 8500-8505, (2014).
5 Mouritsen, O. G. & Bloom, M. Mattress model of lipid-protein interactions in membranes. *Biophys J* **46**, 141-153, (1984).
6 Sodt, A. J., Pastor, R. W. & Lyman, E. Hexagonal Substructure and Hydrogen Bonding in Liquid-Ordered Phases Containing Palmitoyl Sphingomyelin. *Biophys J* **109**, 948-955, (2015).
7 Krogh, A., Larsson, B., von Heijne, G. & Sonnhammer, E. L. Predicting transmembrane protein topology with a hidden Markov model: application to complete genomes. *Journal of molecular biology* **305**, 567-580, (2001).
8 Sperotto, M. M. & Mouritsen, O. G. Dependence of lipid membrane phase transition temperature on the mismatch of protein and lipid hydrophobic thickness. *European biophysics journal : EBJ* **16**, 1-10, (1988).
9 Levental, I. *et al.* Palmitoylation regulates raft affinity for the majority of integral raft proteins. *Proc Natl Acad Sci U S A* **107**, 22050-22054, (2010).
10 Lewis, M. & Rees, D. C. Fractal surfaces of proteins. *Science* **230**, 1163-1165, (1985).
11 Sanner, M. F., Olson, A. J. & Spehner, J. C. Reduced surface: an efficient way to compute molecular surfaces. *Biopolymers* **38**, 305-320, (1996).
